# Sensitive Electrochemical Detection of Caffeic Acid in Wine Based on Fluorine-Doped Graphene Oxide

**DOI:** 10.3390/s19071604

**Published:** 2019-04-03

**Authors:** Venkatesh S. Manikandan, Boopathi Sidhureddy, Antony Raj Thiruppathi, Aicheng Chen

**Affiliations:** 1 Electrochemical Technology Centre, Department of Chemistry, University of Guelph, 50 Stone Road E, Guelph, ON N1G 2W1, Canada; vmanikan@lakeheadu.ca (V.S.M.); bsidhure@uoguelph.ca (B.S.); athirupp@uoguelph.ca (A.R.T.); 2 Department of Chemistry, Lakehead University, 955 Oliver Road, Thunder Bay, ON P7B 5E1, Canada

**Keywords:** fluorinated graphene oxide, caffeic acid, beverage quality control, differential pulse voltammetry, electrochemical sensor

## Abstract

We report here a novel electrochemical sensor developed using fluorine-doped graphene oxide (F-GO) for the detection of caffeic acid (CA). The synthesized graphene oxide (GO) and F-GO nanomaterials were systematically characterized with a scanning electron microscope (SEM), and the presence of semi-ionic bonds was confirmed in the F-GO using X-ray photoelectron spectroscopy. The electrochemical behaviours of bare glassy carbon electrode (GCE), F-GO/GCE, and GO/GCE toward the oxidation of CA were studied using cyclic voltammetry (CV), and the results obtained from the CV investigation revealed that F-GO/GCE exhibited the highest electrochemically active surface area and electrocatalytic activity in contrast to the other electrodes. Differential pulse voltammetry (DPV) was employed for the analytical quantitation of CA, and the F-GO/GCE produced a stable oxidation signal over the selected CA concentration range (0.5 to 100.0 μM) with a low limit of detection of 0.018 μM. Furthermore, the acquired results from the selectivity studies revealed a strong anti-interference capability of the F-GO/GCE in the presence of other hydroxycinnamic acids and ascorbic acid. Moreover, the F-GO/GCE offered a good sensitivity, long-term stability, and an excellent reproducibility. The practical application of the electrochemical F-GO sensor was verified using various brands of commercially available wine. The developed electrochemical sensor successfully displayed its ability to directly detect CA in wine samples without pretreatment, making it a promising candidate for food and beverage quality control.

## 1. Introduction

Caffeic acid (3,4-dihydroxycinnamic acid) is an important compound in the classification of phenolic acids, which exists in certain vegetables (e.g., cabbage, cauliflower, and kale) and fruits (e.g., strawberries, grapes, and apples). It serves as an antioxidant, anti-inflammatory, antibacterial, and immune-modulating agent [1,2,3,4]. The molecular structure of CA is comprised of two hydroxyl groups adjacent to an aromatic ring, which provocatively contributes to its distinctive antioxidative nature [5,6]. Copious amounts of CA derivatives are present in wine and are accountable for maintaining colour, thus protecting the alcoholic beverage from oxidative deterioration [7,8]. Fascinatingly, scientific studies have reported that CA functions as an antitumour agent and may have the ability to lower the impacts of diabetes and cancer [9,10,11,12]. Numerous methods, such as liquid and gas chromatography, capillary electrophoresis, and spectrophotometry, have the capacity to determine phenolic acids and CA derivatives in foods and beverages [13,14]. Although these analytical instruments are advanced, their operation requires skilled personnel and involves multistep sample treatments and testing procedures. Consequently, a cost-effective and efficacious method for the detection of CA is imperative to analyze and assure the quality of the wine. One of the major challenges with CA detection is the interference from other polyphenolic compounds (*p*-coumaric acid, hydroquinone, trans-ferulic acid, and gallic acid) present in wine. Therefore, the development of sensitive and selective electrochemical sensors for the quantitative detection of CA is essential.

Several previously reported works have focused on the quantitative determination of CA utilizing different types of nanostructured materials in various electrolyte media [15,16,17,18]. Fernandes et al. developed a multicomponent electrochemical biosensor for the detection and quantification of CA in white wine. This biosensor was developed through the immobilization of green beans in a chitin matrix, which was subsequently incorporated into a carbon paste electrode. The CA content of white wine was determined using square wave voltammetry, and the biosensor demonstrated a good selectivity [19]. Although redox mediator-based biosensors may have a good selectivity, they have disadvantages including reproducibility and long-term stability [20]. Santos and coworkers fabricated an electrochemical sensor by modifying a glassy carbon electrode (GCE) with a poly(glutamic acid) (PG) film for the electrochemical detection and quantification of CA in red wine [21]. Recently, Thangavelu and coworkers reported on the electrochemical determination of CA using gold and palladium decorated with graphene (Au/Pd/Gr) for the sensitive detection of CA [22]. Yue and coworkers developed an electrochemical sensor using Pd–Au/PEDOT/graphene nanoparticles and investigated its performance for the determination of CA [23]. As noble metals are expensive, carbon-based electrochemical sensors offer an inexpensive route toward the quantification of CA. Graphene and its derivatives are widely used in biological applications, electrochemical sensing, and energy storage due to their remarkable physicochemical properties [24,25,26,27,28,29,30,31,32,33,34,35,36]. Array-based sensing approaches (nose/tongue strategies) using graphene oxide have been reported for the detection of biomatrices and organic molecules [37,38,39]. Chemically/electrochemically reduced graphene oxide (GO) and nitrogen-doped carbon electrochemical sensors were explored for CA detection [40,41,42].

The doping of graphene and GO with heteroatoms (e.g., F, Cl, N, B, and S) at the molecular and atomic levels favourably enhances their electrochemical behaviour and electrocatalytic properties while altering their electronic and physicochemical properties [43,44,45]. Based on the nature of the dopants and their bonding conformations, the characteristics of graphene may improve and be advantageous for desired applications. As a result, the heteroatom-doped graphene derivatives have been explored for their capability as an efficient electrocatalyst for oxygen reduction, energy storage, and supercapacitor applications [46,47,48]. F-GO and its derivatives have invigorated intense research activity due to their exceptional properties (e.g., resistance to high temperature, improved electrocatalytic activity, and outstanding chemical inertness) [49,50,51,52]. In contrast to carbon, fluorine possesses a higher electronegativity, which may bring about attributes such as ionic, semi-ionic, and covalent bonding [53,54]. Further, the electronic structure of F-GO is dissimilar to that of graphene oxide. The doping of graphene with F alters its electronic structure due to the incorporation of sp3 carbon into graphene’s sp2 honeycomb structure [55]. This alteration in its electronic structure might be explained by the electron withdrawing and electron donating nature of fluorine atoms due to their strong electronegativity and the presence of lone-pair electrons [56]. Consequently, fluorine-based graphene derivatives are useful across a range of applications, such as supercapacitors, batteries, metal-free electrocatalyst, and biomedical devices [57,58,59,60,61,62].

The aims of the present study were two-fold: (i) to compare the electrochemical properties of GO and F-GO as an advanced electrochemical sensing material and (ii) to develop a high-performance electrochemical sensor for the sensitive detection of CA. To the best of our knowledge, this is the first report on a highly sensitive and selective F-GO-based electrochemical sensor for the direct measurement of CA in different brands of wine.

## 2. Materials and Methods

### 2.1. Reagents

Caffeic acid, potassium ferrocyanide, potassium chloride, chloroauric acid, and other reagents were purchased from Sigma Aldrich. All reagents used for the experiments were of analytical grade. All solutions used for the experiments were prepared using deionized water (18.2 MΩ cm, Nanopure^®^ diamond™ UV water purification system), and all glassware was thoroughly washed and dried prior to each analysis. Four different brands of red wine were purchased from a liquor retail store. All experiments were conducted using a Britton–Robinson (B-R) electrolyte medium [63]. The BR buffer solution was prepared from a mixture of Phosphoric acid, Glacial acetic acid, and Boric acid at a concentration of 0.1 M, and the pH values of the electrolyte solutions were adjusted with a sodium hydroxide solution (0.5 M).

### 2.2. Electrode Preparation and Modification

The F-GO was synthesized via an improved Hummers’ method with some modifications [64]. Initially, a mixture was prepared by blending 1.0 g graphite, 90.0 mL sulfuric acid (H_2_SO_4_), 10.0 mL orthophosphoric acid (H_3_PO_4_), and 20 mL hydrogen fluoride (HF). Secondly, the prepared mixture was stirred vigorously for 2 h, and the temperature was maintained at 50 °C. Approximately 4.5 g of KMnO_4_ was slowly added to this blend and stirred constantly for another 15 h. Thirdly, the reaction mixture was supplemented with 100 mL of ice, and after a short while, 5.0 mL of 30% H_2_O_2_ was also added. Subsequently, the obtained final product (F-GO) was separated and thoroughly rinsed with 30% hydrochloric acid (HCl), pure water, ethanol, and diethyl ether. Lastly, the resulting F-GO solid was transferred to an oven at 50 °C for drying.

The prepared F-GO was mixed with water (4 mg/mL) and ultrasonicated for 1 h to ensure thorough mixing. A GCE was polished with a 1.0 and 0.3 μm alumina slurry, then rinsed with ethanol and water, and ultrasonicated for 10 min. The 5.0 μL of the F-GO ink was drop cast onto the surface of GCE (dia. Φ = 3 mm; 0.071 cm^2^) and air dried for 4 h to obtain the F-GO/GCE. The as-prepared F-GO/GCE was then subjected to a partial electrochemical reduction in a 0.1 M phosphate buffer solution at pH 7.4. Similarly, another GCE was prepared by drop-casting 5.0 μL of the GO solution onto its surface. For comparison, a bare GCE and the GO/GCE were tested alongside with F-GO/GCE for CA oxidation [56].

### 2.3. Characterization Techniques

The F-GO was characterized using a Hitachi SU-70 Schottky Field Emission SEM with an energy dispersive X-ray spectrometer and XPS (Thermo Scientific). XPS was carried out using a Thermo Scientific K-α XPS spectrometer, and the spectra was fitted by the XPS PEAK software. A monochromatic Al Kα X-ray source was employed with a spot size of 400 μm. Voltammetry techniques such as CV and DPV were implemented using a CHI potentiostat (CHI-660D, CHI, USA). The redox behaviour of the molecule and the kinetics of the electrodes were examined using CV, whereas the analytical determination of the electroactive analyte was conducted by means of DPV. All electrochemical experiments were conducted in an electrochemical cell consisting of a conventional three-electrode system. The F-GO/GCE functioned as the working electrode, a coiled platinum wire acted as the counter electrode, and a standard silver–silver chloride (3 M KCl saturated Ag/AgCl) electrode served as the reference electrode. All analytical quantitations were performed in a 0.1 M B-R buffer (pH 2.65) at room temperature (22 ± 2 °C). Preceding the analysis, all of the solutions were purged with argon gas for a duration of 15 min. The potentials specified in this paper were against an Ag/AgCl reference electrode.

## 3. Results and Discussion

### 3.1. Morphology and Electrochemical Characterization

**Figure 1:**
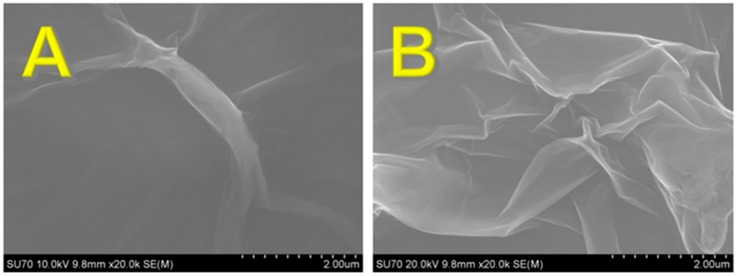
SEM images of the synthesized (**A**) graphene oxide (GO) and (**B**) fluorinated graphene oxide (F-GO).

The surface morphologies of the F-GO and GO were investigated using SEM as displayed in Figure S1 (low magnification) and Figure 1 (high magnification). Figure 1A presents a typical SEM image observed for the GO, and Figure 1B illustrates the SEM image for the F-GO, which revealed a crumpled layered structure. In addition, XPS was utilized to distinguish the composition and functional group species that were present in the F-GO and GO. Gaussian and Lorentzian functions were used to deconvolute the peaks in the high resolution XPS spectra, and the sp^2^ carbon was fitted at 284.5 eV [65].

**Figure 2:**
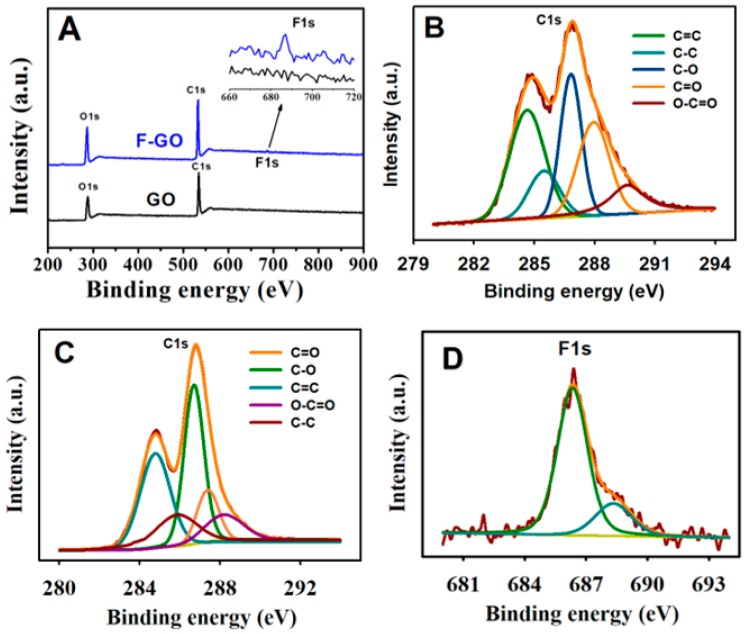
(**A**) The XPS spectra (survey scan) of GO and FGO; (**B**) high-resolution XPS spectrum of C1s of GO and the fitting curves; (**C**) high-resolution XPS spectrum of C1s of F-GO and the fitting curves; and (**D**) high-resolution XPS spectrum of F1s of F-GO.

As perceived from the expanded inset of the XPS survey scans (Figure 2A), the presence of fluorine was verified by the occurrence of the F peak at approx. 686.75 eV. The percentages of C, O, and F analyzed from the data of F-GO were 65.20%, 33.64%, and 1.16%, respectively. In contrast, the calculation from the GO composition data revealed the percentages of C and O to be 65.17% and 34.83%, respectively. The high-resolution C1s spectra of GO and F-GO were deconvoluted into five distinct peaks (Figure 2B,C). The five distinct peaks from the C1s spectrum of GO were as follows: C=C (284.7 eV), C–C (285.4 eV), C–O (286.8 eV), C=O (287.9 eV), and O–C=C (289.2 eV).

Prominent differences were observed in terms of the peak intensities when the C1s spectrum F-GO was equated with that of GO. The peak heights of C=O and O–C=O displayed decreased F-GO intensity, whereas a higher intensity of C–O peak was observed, which was attributed to the attack of F on the C=O group and to the formation of C–O and C–F bonds. Further validation was supplemented by the use of the high-resolution F1s spectrum of F-GO (Figure 2D). It may be observed from the spectrum that the main peak appeared at 686.38 eV, which was ascribed to the semi-ionic nature of the C–F bond, and the shoulder peak appearing at 689.15 eV, parallel to the C–F bond, which was covalent in nature.

**Figure 3:**
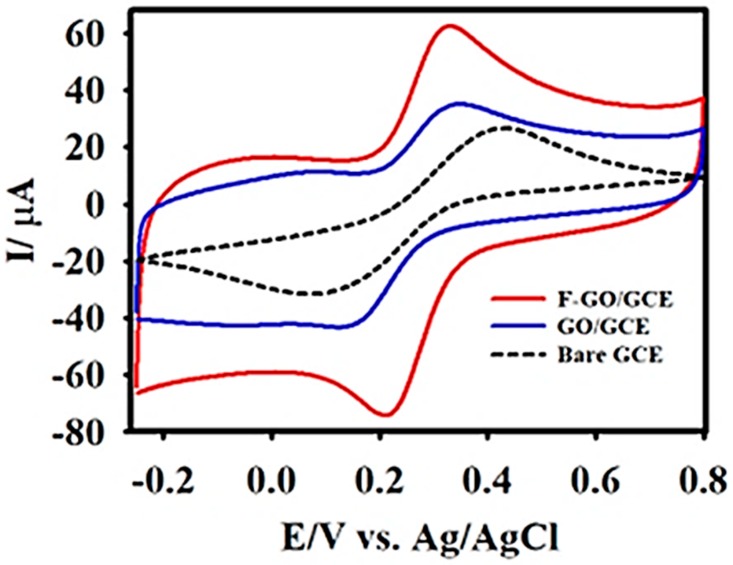
The cyclic voltammetry (CV) curves of the bare glassy carbon electrode (GCE) (black dashed line), GO/GCE (blue line), and F-GO/GCE (red line) recorded in 0.1 M KCl containing 5.0 mM [Fe(CN)_6_]^3−/4−^ at the scan rate of 50 mV s^−1^.

The electrochemical characteristics of the bare GCE, GO/GCE, and F-GO/GCE were examined in a KCl-Ferricyanide medium (5 mM [Fe(CN)_6_]^3−/4−^ in 0.1 M KCl). Figure 3 exhibits the well-defined cyclic voltammetric curves obtained for the oxidation and reduction of 5 mM [Fe(CN)_6_]^3−/4−^ at the bare GCE, GO/GCE, and F-GO/GCE. In contrast to the bare GCE and GO/GCE, the F-GO/GCE demonstrated a higher anodic-cathodic peak current and a smaller peak-to-peak potential separation. The potential separations for the bare GCE, GO/GCE, and F-GO/GCE were calculated to be 294, 158, and 113 mV, respectively. The smaller peak-to-peak separation demonstrated a better electrochemical reversibility and an improved electron transfer efficiency [61].

Furthermore, the value obtained from the ratio of the anodic to cathodic peak current (Ipa/Ipc = 0.96) confirmed that the oxidation-reduction of the [Fe(CN)_6_]^3−/4−^ redox probe at the F-GO/GCE was reversible. Subsequently, Figure S1 shows a series of CV curves obtained at different scan rates (10–100 mV s^−1^) in the KCl-ferricyanide medium to study the redox behaviour of the modified electrode and to calculate the electrochemically active surface area (EASA) of the F-GO/GCE using the Randles–Sevcik equation [66]:

(1) $$ip=2.69\times 10^{5}\;n^{3/2}\;AD^{1/2}\;C\;{ʋ}^{1/2}$$

where *ip* represents the peak current, *D* stands for the diffusion coefficient (cm^2^ s^−1^), *C* denotes the concentration of the [Fe(CN)_6_]^3−/4−^ molecules in mol L^−1^, *A* is the EASA (cm^2^), *n* signifies the electron transfer number, and *ʋ* is the scan rate (V s^−1^). Figure S2 displays the slopes obtained for the Ipa versus ʋ^1/2^ plots; the EASA was calculated to be 0.045, 0.075, and 0.745 cm^2^ for the bare GCE, GO/GCE, and F-GO/GCE, respectively. These observed results verified that the fabricated F-GO/GCE had a higher EASA compared to GO and bare GCE.

### 3.2. Electrooxidation Behaviour of CA

**Figure 4:**
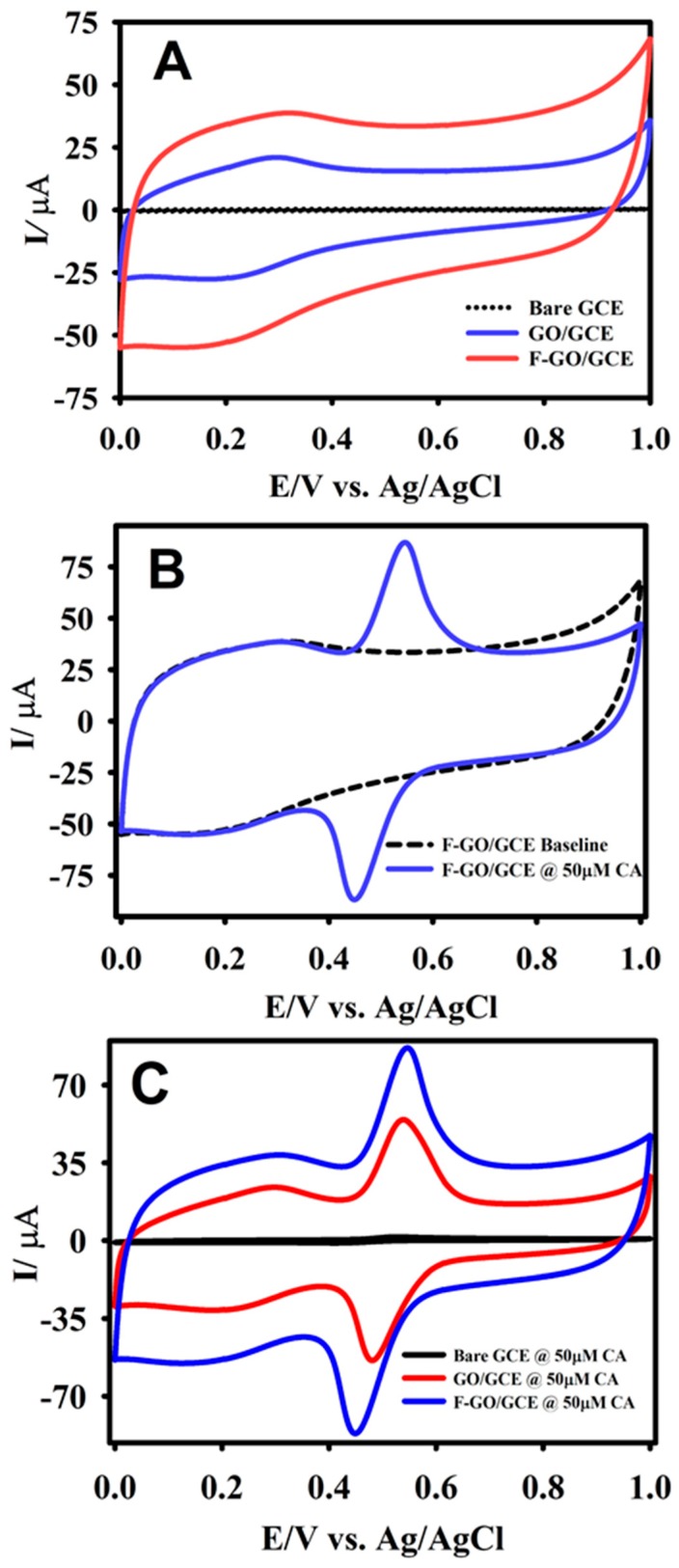
The CV curves of bare GCE (dashed black line), GO/GCE (blue line), and F-GO/GCE (red line) recorded in the absence of caffeic acid (CA) (**A**); the F-GO/GCE response in the presence of 50.0 μM CA (**B**); a comparison of the modified GCEs in the presence of 50.0 μM CA (**C**) in a 0.1 M B-R buffer solution (pH 2.65) at a scan rate of 50 mV s^−1^.

Cyclic voltammetry was primarily employed to investigate the electrooxidation behavior of CA at the F-GO modified GCE. Figure 4A illustrates the response curves for the bare GCE, GO/GCE, and F-GO recorded in a 0.1 M B-R buffer solution at a scan rate of 50 mV s^−1^ in the absence of CA. There were no anodic/cathodic peaks that appeared for the prepared GCEs without CA in the B-R buffer solution. It was evident from the CV that the F-GO/GCE had a larger background current when compared to the bare GCE and GO/GCE, which further confirmed that F-GO had a much larger EASA.

From Figure 4B, it can be seen that the CV displayed well-defined peaks for the oxidation and reduction of 50 µM CA. As shown in Figure 4C, the modified GCEs exhibited a sharp quasi-reversible peak for the oxidation and reduction of 50 µM CA at different peak potentials. The emergence of the redox peaks was due to the formation of o-quinone following CA oxidation. The chemical reaction for the oxidation of CA followed a 2-electron transfer process [41]:

C_9_H_8_O_4_ (CA) ⇋ C_9_H_6_O_4_ + 2 H^+^ + 2 e^−^ (2)

The F-GO/GCE amplified the rate of the electron transfer and enhanced the overall response toward the electrochemical oxidation of CA. The current response recorded with the F-GO/GCE showed a higher anodic and cathodic peak intensity for the oxidation/reduction of CA as opposed to the bare GCE and GO/GCE. The current response of the F-GO/GCE was 1.5 and 50 times higher than that of the GO/GCE and the bare GCE, respectively. The presence of the C–F semi-ionic bond and a higher conductivity facilitated the higher electrocatalytic oxidation of CA. Moreover, the Δ*Ep* values calculated from the CA oxidation/reduction peak potentials with F-GO/GCE (90 mV) were lower than that of the bare GCE (117 mV), further confirming that F-GO/GCE possessed a good electrocatalytic activity toward the oxidation of CA.

### 3.3. Effects of Scan Rate, Electrolyte pH, and Concentration

**Figure 5:**
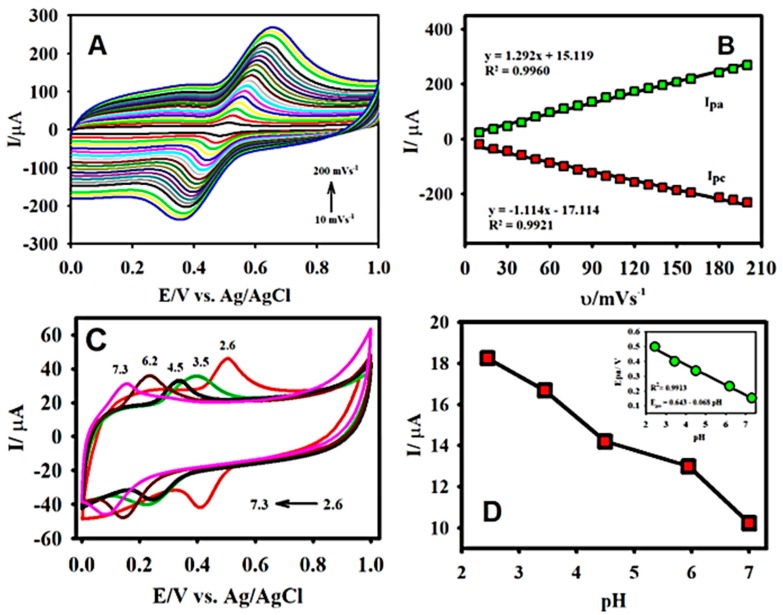
The CV response of F-GO/GCE recorded in the presence of 50 μM CA at different scan rates varying between 10 to 200 mV s^−1^ (**A**) and the subsequent plot of anodic/cathodic peak current vs. the scan rate (mV s^−1^) (**B**); the CV curves for the oxidation of 30.0 µM CA at different pHs (2.6–7.3) (**C**); and the resulting plot of anodic peak current vs pH (**D**) and the corresponding plot of the anodic peak potential vs. pH (insert of D). Electrolyte: 0.1 M B-R buffer solution pH 2.65; scan rate: 50 mV s^−1^.

Based on the effect of the scan rate, the electrocatalytic oxidation of CA was investigated further by employing CV. Figure 5A illustrates the effect of the scan rate on the electrochemical oxidation of 50.0 µM CA at the F-GO/GCE recorded in a 0.1 M B-R buffer from 10 to 200 mV s^−1^. The values of the anodic/cathodic peak current were incremental with the increased scan rates; however, the observations from the CV exhibited a shift in the oxidation/reduction peak potentials, indicating that the electrochemical oxidation/reduction process was quasi-reversible. At higher scan rates, the electrochemical reaction became less reversible due to the slow electron-transfer rate [40,67].

The displaced peak potential could also be attributed to the change of the thickness of the diffusion layer, which varied with the scan rate [68]. The corresponding plot of the oxidation/reduction peak current Ipa/pc vs. the scan rate (ʋ, mV s^−1^) is presented in Figure 5B. The observation from the plot indicated that the CA oxidation exhibited a good linearity over the incrementing scan rate. The estimated R^2^ values and the linear regression equation from the calibration plot were R^2^ = 0.9960, Ipa = 1.292 × 10^−6^ A/mV s^−1^ + 15.119 µA and R^2^ = 0.9921, Ipc = −1.114 × 10^−6^ A/mV s^−1^ − 17.114 µA (Figure 4B).

The estimated R^2^ values and the linear regression equation from the calibration plot were R^2^ = 0.9960, Ipa = 1.292 × 10^−6^ A/mV s^−1^ + 15.119 µA and R^2^ = 0.9921, Ipc = −1.114 × 10^−6^ A/mV s^−1^ − 17.114 µA (Figure 4B). The results from the linear relationship between Ipa/pc and the scan rate indicated that the oxidation and reduction of CA at the F-GO/GCE was a surface-controlled process.

The effect of the electrolyte pH played a vital role in enhancing the electrocatalytic performance of the sensor and in determining the electrochemical oxidation behaviour of the CA molecule. The electrochemical behaviour of CA was investigated by running a CV in the B-R buffer solution under a diverse pH range (acidic to neutral), which contained 30.0 µM of the analyte at a scan rate of 50 mV s^−1^. Figure 5C displays the CV curves of the F-GO/GCE toward the electrochemical oxidation of CA at different pH levels, ranging from 2.6 to 7.3. The peak potential was linearly shifted towards a more negative potential with the increase of the pH as seen in the insert of Figure 5D, which was consistent with the report in the literature [67,69]. On the other hand, the peak current was decreased with the increase of the pH as shown in Figure 5D. As a result, the B-R buffer solution with pH 2.65 was selected for carrying out further electrochemical experiments and for the analytical determination of CA.

**Figure 6:**
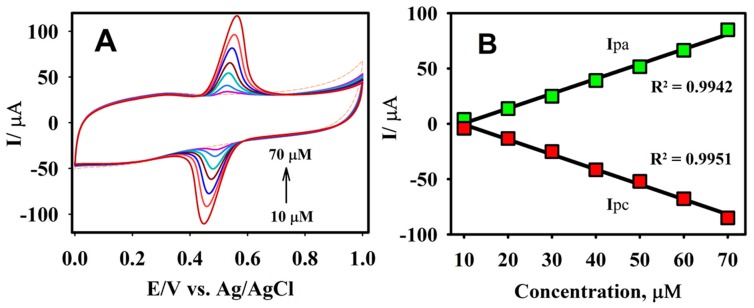
The CV responses of F-GO/GCE for the oxidation/reduction of CA at different concentrations, ranging from 10.0 to 70.0 μM (**A**) and the corresponding plots of the anodic/cathodic peak current vs. the concentration of CA (**B**) in a 0.1 M B-R buffer solution pH 2.65, at a scan rate of 50 mV s^−1^.

The oxidation behavior of CA was further investigated in a 0.1 M B-R buffer solution (pH = 2.65) at a scan rate of 50 mV s^−1^ under different concentrations. It is evident from Figure 6A that there was no apparent peak for the absence of the CA molecule, whereas a clear response was observed for the addition of 10.0 µM CA. The peak current for the oxidation and reduction of CA increased linearly, with each subsequent addition in a selected concentration range from 0.0 to 70.0 µM. The anodic and cathodic peak current values obtained from the CVs were plotted against the various concentrations of CA (Figure 6B). It may be perceived from the calibration plot that there was a good linearity within the selected calibration range, with the linear regression equations of Ipa (μA) = 1.354 μA/μM − 13.61 μA and Ipc = −1.362 μA/μM − 3.06 μA with R^2^ values of 0.9942 for Ipa and 0.9951 for Ipc.

### 3.4. Analytical Determination of CA

**Figure 7:**
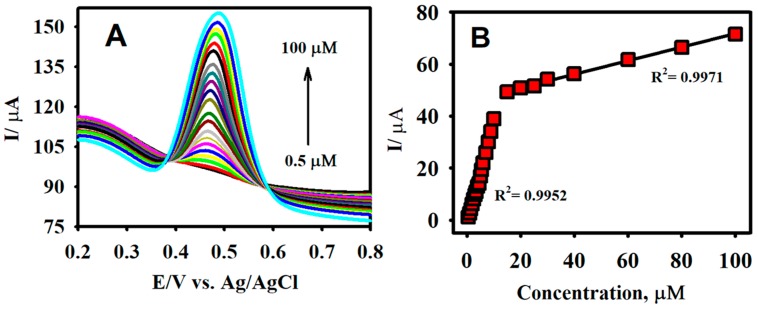
The differential pulse voltammetry (DPV) responses of F-GO/GCE for the detection of CA in the concentration ranging from 0.5 to 100.0 µM (**A**) and the corresponding calibration plot for the anodic peak current vs. concentration (**B**) in a 0.1 M B-R buffer solution (pH 2.65).

The analytical capacity of the F-GO/GCE was investigated by employing DPV. In contrast to CV, DPV is more sensitive and has the ability to lower the background current [70]. Figure 7A illustrates the characteristic DPV responses for different concentrations of CA using the F-GO/GCE electrochemical sensor. The working parameters for the DPV technique were amplitude = 0.08 V, pulse width = 0.08 s, sampling width = 0.0167 s, and pulse period = 0.2 s. The F-GO/GCE did not exhibit any response in the absence of CA, which suggested that the sensor was inactive in the absence of the CA molecule within the selected potential window. However, the DPV responses obtained from the electrochemical oxidation of CA demonstrated a linear increase with respect to its increasing concentration, ranging from 0.5–100.0 μM.

The linear regression equation between the concentration (c, µM) and the current (Ipa, µA) for the electrochemical oxidation of CA in the concentration ranges from 0.5–10.0 μM and 15.0–100.0 μM (Figure 7B) were Ipa = 3.935 c (µM) + 1.919 and Ipa = 0.261 c (µM) + 38.658. The associated R^2^ coefficient values from the calibration plot were calculated to be 0.9960 and 0.9971, respectively. The sensitivities from the calibration plots were calculated to be 5.27 and 0.35 µAµM^−1^ cm^−2^. The limit of detection (LOD) was further calculated to be 0.018 µM using the formula 3 *σ/s*, where *σ* denotes the standard deviation of three blank measurements and *s* represents the slope calculated from the calibration plot. The two calibration plots acquired in this study were similar to studies that involved the measurement of molecules, such as acetaminophen and valacyclovir, ascorbic acid, dopamine, uric acid, and NADH [25,59,71,72]. This observation might be explained based on the mechanism of the inner-sphere electrode reaction [25,73]. Important attributes such as the novelty analytical advantages and capabilities (e.g., linear range and LOD) of the developed F-GO/GCE electrochemical sensor were confirmed by comparing the performance of the CA sensors formerly reported in the literature as shown in Table 1.

### 3.5. Stability, Reproducibility, and Interference Measurements

**Figure 8:**
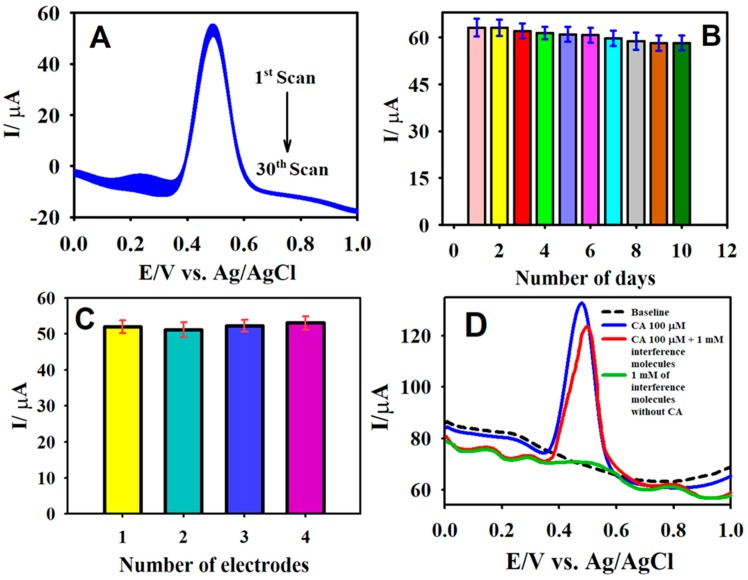
The DPV curves of the F-GO/GCE recorded in 50.0 µM CA over 30 consecutive measurements (**A**); the DPV response of F-GO/GCE measured in 50.0 µM CA for 10 days (**B**); the DPV response recorded in the presence of 50.0 µM CA with four different F-GO/GCEs (**C**); and the interference response of the F-GO/GCE for the detection of 100.0 µM CA in the presence of 1.0 mM *p*-coumaric acid, hydroquinone, trans-ferulic acid, gallic acid, glucose, and ascorbic acid (**D**). Electrolyte: a 0.1 M B-R buffer solution (pH 2.65).

The stability of the sensor was scrutinized by employing DPV for 30 consecutive scans (Figure 8A). The sensor retained 94.7% of its initial peak current response for a 50.0 μM CA oxidation in a 0.1 M B-R buffer medium (pH 2.65). In addition, the long-term stability of the fabricated sensor was tested over ten days, where the sensor preserved 95% of its primary response with a relative standard deviation (RSD) value of 2.41%. This demonstrated the good stability of the sensor and could be applied to the evaluation of actual samples (Figure 8B). Further, the reproducibility of the F-GO/GCE was inspected via the preparation of four different electrodes. The reproducibility tests were carried out in 0.1 M PBS containing 50.0 µM CA under identical experimental conditions. The RSD value for the anodic peak current measured at the four different electrodes was calculated to be 2.1%, thereby indicating the excellent reproducibility of the F-GO/GCE sensor.

The selectivity of the developed electrochemical sensor was an essential factor for the detection of CA to enable the testing of actual samples. Henceforth, the prepared F-GO/GCE sensor was tested for its anti-interference capabilities in the presence of potentially interfering species using DPV. Figure 8D illustrates the DPV response recorded for the oxidation of 100.0 µM in the presence of a 10-fold (1.0 mM) concentration of interfering ions, such as *p*-coumaric acid, hydroquinone, trans-ferulic acid, gallic acid, glucose, and ascorbic acid. The results obtained from the interference study revealed that the F-GO/GCE sensor retained 92% of its activity for 100.0 µM CA oxidation in the presence of interference molecules. Therefore, the developed F-GO/GCE electrochemical sensor could be applied to the real-time analysis of CA.

### 3.6. Real Sample Analysis

The electrochemical performance of the designed F-GO/GCE sensor was further validated for its real-time determination of CA in wine. Four different samples of red wine were used for the determination of CA. The wine was tested by injecting raw samples directly into the 0.1 M B-R buffer solution, and the concentrations of CA were determined from their respective DPV peaks. The DPV peaks obtained for the determination of CA in wine samples (50.0 µL to 300.0 µL) are included in the supplementary information (Figure S4A–D). The peak current values from DPV toward CA oxidation for the injected volume of the wine sample was compared against the calibration plot, thereby estimating the unknown concentration of CA. The concentration of CA found in four different wine samples was quantified in the range from 73.4–94.5 µM, with an average RSD value of 2.3%. A further high-performance liquid chromatography (HPLC, Agilent) analysis showed that the CA concentration in Wine Sample II was 77.5 µM, which is consistent with the value measured by the F-GO electrochemical sensor.

The exact procedure was followed for analyzing the concentrations of CA in other wine samples, and the results of the quantification of CA in the assorted brands of wine are presented in Table 2. The wine samples were diluted with the B-R electrolyte medium. Based on the constructed calibration plot using DPV, the amount of CA in the commercial red wine was calculated, which were comparable with the results reported in the literature [42,79,80]. Furthermore, the fabricated electrochemical sensor was able to detect CA directly in wine (wine as the electrolyte) without the use of a buffer solution (Figure S5). These results suggest that the developed F-GO/GCE sensor could be effectively used for the determination of CA in wine and has real-time applicability.

## 4. Conclusions

A highly selective F-GO based sensor was developed for the sensitive detection of CA in assorted brands of wine. The results obtained from the CV investigations indicated that the F-GOs enhanced the electrocatalytic behaviour in contrast to bare GCE and GO modified GCE. The F-GO/GCE exhibited a higher EASA and improved the electrochemical performance as opposed to the GO/GCE. The analytical quantitation performed using DPV revealed the strong capability of the electrochemical sensor for the determination of CA within a wide linear concentration range in a 0.1 M B-R buffer (pH 2.65). The F-GO/GCE sensor demonstrated a superior selectivity for the oxidation of CA in the presence of additional phenolic compounds. The developed electrochemical sensor exhibited a good long-term stability and sensitivity, a low detection limit, and a satisfactory reproducibility. Further, it was effectively employed for the detection and quantification of CA in various brands of red wine. The sensor also displayed a superior sensing capacity to detect CA directly in wine without the requirement of an external electrolyte medium. These collective factors confirmed that the developed F-GO/GCE sensor could be incorporated for beverage quality control.

## Figures and Tables

**Table 1:** A comparison of the electrochemical performance of the F-GO/GCE sensor with other reported sensors for the detection of CA.

Modified Electrode	Voltammetric Technique	Linear Range (µM)	Limit of Detection (µM)	Ref.
Molecularly imprinted siloxanes	DPV	0.5–60.0	0.15	[74]
Electrochemically reduced graphene oxide: Nafion	SWAdSV	0.1–10.0	0.09	[40]
Laccase-MWCNT-chitosan/Au	Amperometric	0.7–10.0	0.15	[75]
Nafion/Tyre/Sonogel-Carbon	Amperometric	0.08–2.0	0.06	[76]
Glassy polymeric carbon	DPV	0.1–96.5	0.29	[77]
Glassy carbon electrode	DPV	10.0–120.0	0.10	[78]
Poly(Glutamic Acid)/GCE	CV	4.0–30.0	3.91	[21]
Fluorine doped graphene oxide/GCE	DPV	0.5–100.0	0.018	This work

DPV, Differential pulse voltammetry, SWAdSV, Square wave anodic stripping voltammetry, CV, Cyclic voltammetry, MWCNT, Multi-walled carbon nanotubes, GCE, Glassy carbon electrode.

**Table 2:** The performance of the F-GO/GCE sensor for the determination of CA in different brands of wine.

Red Wine Sample	Found (µM)	% RSD (n = 3)
Wine sample I	74.4	2.3
Wine sample II	84.3	2.0
Wine sample III	85.1	2.2
Wine sample IV	94.5	2.1

## Supplementary Materials

The following are available online at https://www.mdpi.com/1424-8220/19/7/1604/s1 Figure S1: SEM images (low magnification) of the synthesized (A) graphene oxide (GO) and (B) fluorinated graphene oxide (F-GO); Figure S2: (A) The CV curves of the F-GO/GCE recorded in the presence of 5 mM [Fe(CN)_6_]^3−/4−^ in 0.1 M KCl at various scan rates, ranging from 10–100 mV s^−1^ and (B) the corresponding plots vs the anodic/cathodic peak current vs the square root of the scan rates; Figure S3: The DPV response of F-GO/GCE recorded in the presence of 50.0 µM CA (blue line) and in the absence of CA (black dashed line) (A) and a comparison of the DPV response of bare GCE (blue dashed line), GO/GCE (green line), and F-GO/GCE (red line) recorded in the presence of 50.0 µM CA in a 0.1 M B-R buffer solution (pH 2.65); Figure S4: The DPV response obtained with F-GO/GCE for the detection of CA in assorted brands of red wine in a 0.1 M B-R buffer solution (pH 2.65); Figure S5: The DPV response of the F-GO/GCE sensor toward the detection of CA directly in the wine sample without an electrolyte medium.

## References

[1] Li J., Jiang J., Liu M., Xu Z., Deng P., Qian D., Tong C., Xie H., Yang C. (2017). Facile synthesis of MnO_2_-embedded flower-like hierarchical porous carbon microspheres as an enhanced electrocatalyst for sensitive detection of caffeic acid. Anal. Chim. Acta.

[2] Xu X., Xu G., Wei F., Cen Y., Shi M., Cheng X., Chai Y., Sohail M., Hu Q. (2018). Carbon dots coated with molecularly imprinted polymers: A facile bioprobe for fluorescent determination of caffeic acid. J. Colloid Interface Sci..

[3] Moreira G.C., de Souza Dias F. (2018). Mixture design and Doehlert matrix for optimization of the ultrasonic assisted extraction of caffeic acid, rutin, catechin and trans-cinnamic acid in Physalis angulata L. and determination by HPLC DAD. Microchem. J..

[4] García-Guzmán J.J., López-Iglesias D., Cubillana-Aguilera L., Lete C., Lupu S., Palacios-Santander J.M., Bellido-Milla D. (2018). Assessment of the polyphenol indices and antioxidant capacity for beers and wines using a tyrosinase-based biosensor prepared by sinusoidal current method. Sensors.

[5] Bo Z., Shuai X., Mao S., Yang H., Qian J., Chen J., Yan J., Cen K. (2014). Green preparation of reduced graphene oxide for sensing and energy storage applications. Sci. Reports.

[6] Ramki S., Balasubramanian P., Chen S.-M., Chen T.-W., Tseng T.-W., Lou B.-S. (2018). Voltammetric determination of caffeic acid using Co_3_O_4_ microballs modified screen-printed carbon electrode. Int. J. Electrochem. Sci.

[7] Robbins R.J. (2003). Phenolic acids in foods: An overview of analytical methodology. J. Agric. Food Chem..

[8] Alanko J., Riutta A., Holm P., Mucha I., Vapaatalo H., Metsä-Ketelä T. (1999). Modulation of arachidonic acid metabolism by phenols: Relation to their structure and antioxidant/prooxidant properties. Free Radic. Biol. Med..

[9] Kang N.J., Lee K.W., Shin B.J., Jung S.K., Hwang M.K., Bode A.M., Heo Y.-S., Lee H.J., Dong Z. (2009). Caffeic acid, a phenolic phytochemical in coffee, directly inhibits fyn kinase activity and UVB-induced CO_X_-2 expression. Carcinogenesis.

[10] Nardini M., Scaccini C., Packer L., Virgili F. (2000). In vitro inhibition of the activity of phosphorylase kinase, protein kinase C and protein kinase A by caffeic acid and a procyanidin-rich pine bark (Pinus marittima) extract. Biochim. Biophys. Acta (BBA) Gen. Subj..

[11] Robards K., Antolovich M. (1997). Analytical chemistry of fruit bioflavonoids A Review. Analyst.

[12] Hsu F.-L., Chen Y.-C., Cheng J.-T. (2000). Caffeic acid as active principle from the fruit of xanthiumstrumarium to lower plasma glucose in diabetic rats. Planta Med..

[13] Youyuan P., Fanghua L., Jiannong Y. (2005). Determination of phenolic acids and flavones in lonicera japonica thumb. by capillary electrophoresis with electrochemical detection. Electroanal..

[14] Manikandan V.S., Adhikari B., Chen A. (2018). Nanomaterial based electrochemical sensors for the safety and quality control of food and beverages. Analyst.

[15] Zeng C.-C., Liu C.-F., Zeng J., Zhong R.-G. (2007). Electrochemical synthesis of 6-arylsulfonyl caffeic acid derivatives in aqueous medium. J. Electroanal. Chem..

[16] Makhotkina O., Kilmartin P.A. (2009). Uncovering the influence of antioxidants on polyphenol oxidation in wines using an electrochemical method: Cyclic voltammetry. J. Electroanal. Chem..

[17] Sivasankar K., Devasenathipathy R., Wang S.-F., Kohila rani K., Raja D.S., Lin C.-H. (2018). Synthesis of hierarchical mesoporous graphite oxide/Al_2_O_3_ from MIL-100(Al) for the electrochemical determination of caffeic acid in red wine samples. J. Taiwan Inst. Chem. E..

[18] Gao L., Yue R., Xu J., Liu Z., Chai J. (2018). Pt-PEDOT/rGO nanocomposites: One-pot preparation and superior electrochemical sensing performance for caffeic acid in tea. J. Electroanal. Chem..

[19] Fernandes S.C., de Oliveira I.R.W.Z., Vieira I.C. (2007). A green bean homogenate immobilized on chemically crosslinked chitin for determination of caffeic acid in white wine. Enzyme Microb. Technol..

[20] Yao Y., Wen Y., Zhang L., Wang Z., Zhang H., Xu J. (2014). Electrochemical recognition and trace-level detection of bactericide carbendazim using carboxylic group functionalized poly(3,4-ethylenedioxythiophene) mimic electrode. Anal. Chim. Acta.

[21] Santos D.P., Bergamini M.F., Fogg A.G., Zanoni M.V.B. (2005). Application of a glassy carbon electrode modified with Poly(glutamic acid) in caffeic acid determination. Microchim. Acta.

[22] Thangavelu K., Raja N., Chen S.-M., Liao W.-C. (2017). Nanomolar electrochemical detection of caffeic acid in fortified wine samples based on gold/palladium nanoparticles decorated graphene flakes. J. Colloid Interface Sci..

[23] Liu Z., Lu B., Gao Y., Yang T., Yue R., Xu J., Gao L. (2016). Facile one-pot preparation of Pd-Au/PEDOT/graphene nanocomposites and their high electrochemical sensing performance for caffeic acid detection. RSC Adv..

[24] Taghioskoui M. (2009). Trends in graphene research. Mater. Today.

[25] Adhikari B.-R., Govindhan M., Schraft H., Chen A. (2016). Simultaneous and sensitive detection of acetaminophen and valacyclovir based on two-dimensional graphene nanosheets. J. Electroanal. Chem..

[26] Adhikari B.-R., Govindhan M., Chen A. (2015). Carbon nanomaterials based electrochemical sensors/biosensors for the sensitive detection of pharmaceutical and biological compounds. Sensors.

[27] Adhikari B.-R., Govindhan M., Chen A. (2015). Sensitive detection of acetaminophen with graphene-based electrochemical sensor. Electrochim. Acta.

[28] Shah B., Lafleur T., Chen A. (2013). Carbon nanotube based electrochemical sensor for the sensitive detection of valacyclovir. Faraday Discuss..

[29] Govindhan M., Lafleur T., Adhikari B.R., Chen A. (2015). Electrochemical sensor based on carbon nanotubes for the simultaneous detection of phenolic pollutants. Electroanalysis.

[30] Cheng C., Li S., Thomas A., Kotov N.A., Haag R. (2017). Functional graphene nanomaterials-based architectures: Biointeractions, fabrications, and emerging biological applications. Chem. Rev..

[31] Mapasha R.E., Igumbor E., Andriambelaza N.F., Chetty N. (2018). Electronic properties of B and Al doped graphane: A hybrid density functional study. Phys. B Condens. Matter..

[32] Baghayeri M. (2017). Pt nanoparticles/reduced graphene oxide nanosheets as a sensing platform: Application to determination of droxidopa in presence of phenobarbital. Sens. Actuators B Chem..

[33] Hernaez M., Zamarreño C.R., Melendi-Espina S., Bird L.R., Mayes A.G., Arregui F.J. (2017). Optical fibre sensors using graphene-based materials: A review. Sensors.

[34] Huang J., Yang X., Her S.-C., Liang Y.-M. (2019). Carbon nanotube/graphene nanoplatelet hybrid film as a flexible multifunctional sensor. Sensors.

[35] Suvarnaphaet P., Pechprasarn S. (2017). Graphene-based materials for biosensors: A review. Sensors.

[36] Chen K., Zhang Z.-L., Liang Y.-M., Liu W. (2013). A graphene-based electrochemical sensor for rapid determination of phenols in water. Sensors.

[37] Wu L., Ji H., Guan Y., Ran X., Ren J., Qu X. (2017). A graphene-based chemical nose/tongue approach for the identification of normal, cancerous and circulating tumor cells. NPG Asia Mater..

[38] Chou S.S., De M., Luo J., Rotello V.M., Huang J., Dravid V.P. (2012). Nanoscale graphene oxide (nGO) as artificial receptors: Implications for biomolecular interactions and sensing. J. Am. Chem. Soc..

[39] Cavallari M.R., Braga G.S., da Silva M.F., Izquierdo J.E., Paterno L.G., Dirani E.A., Kymissis I., Fonseca F.J. (2017). A hybrid electronic nose and tongue for the detection of ketones: Improved sensor orthogonality using graphene oxide-based detectors. IEEE Sens. J..

[40] Filik H., Çetintaş G., Avan A.A., Aydar S., Koç S.N., Boz İ. (2013). Square-wave stripping voltammetric determination of caffeic acid on electrochemically reduced graphene oxide–nafion composite film. Talanta.

[41] Ezhil Vilian A.T., Chen S.-M., Chen Y.-H., Ajmal Ali M., Al-Hemaid F.M.A. (2014). An electrocatalytic oxidation and voltammetric method using a chemically reduced graphene oxide film for the determination of caffeic acid. J. Colloid Interface Sci..

[42] Karikalan N., Karthik R., Chen S.-M., Chen H.-A. (2017). A voltammetric determination of caffeic acid in red wines based on the nitrogen doped carbon modified glassy carbon electrode. Sci. Rep..

[43] Georgakilas V., Otyepka M., Bourlinos A.B., Chandra V., Kim N., Kemp K.C., Hobza P., Zboril R., Kim K.S. (2012). Functionalization of graphene: Covalent and non-covalent approaches, derivatives and applications. Chem. Rev..

[44] Chronopoulos D.D., Bakandritsos A., Pykal M., Zbořil R., Otyepka M. (2017). Chemistry, properties, and applications of fluorographene. Appl. Mater. Today.

[45] Ji H., Zhou F., Gu J., Shu C., Xi K., Jia X. (2016). Nitrogen-doped carbon dots as a new substrate for sensitive glucose determination. Sensors.

[46] Zheng B., Wang J., Wang F.-B., Xia X.-H. (2013). Synthesis of nitrogen doped graphene with high electrocatalytic activity toward oxygen reduction reaction. Electrochem. Commun..

[47] Vizintin A., Lozinšek M., Chellappan R.K., Foix D., Krajnc A., Mali G., Drazic G., Genorio B., Dedryvère R., Dominko R. (2015). Fluorinated reduced graphene oxide as an interlayer in li–s batteries. Chem. Mater..

[48] Han J., Zhang L.L., Lee S., Oh J., Lee K.-S., Potts J.R., Ji J., Zhao X., Ruoff R.S., Park S. (2013). Generation of b-doped graphene nanoplatelets using a solution process and their supercapacitor applications. ACS Nano.

[49] Jeon K.-J., Lee Z., Pollak E., Moreschini L., Bostwick A., Park C.-M., Mendelsberg R., Radmilovic V., Kostecki R., Richardson T.J. (2011). Fluorographene: A wide bandgap semiconductor with ultraviolet luminescence. ACS Nano.

[50] Cheng L., Jandhyala S., Mordi G., Lucero A.T., Huang J., Azcatl A., Addou R., Wallace R.M., Colombo L., Kim J. (2016). Partially Fluorinated Graphene: Structural and Electrical Characterization. ACS Appl. Mater. Interfaces.

[51] Wang X., Sun G., Routh P., Kim D.-H., Huang W., Chen P. (2014). Heteroatom-doped graphene materials: Syntheses, properties and applications. Chem. Soc. Rev..

[52] Withers F., Dubois M., Savchenko A.K. (2010). Electron properties of fluorinated single-layer graphene transistors. Phys. Rev. B.

[53] Wei F., Peng L., Yiyu F., Yu L. (2016). Two-Dimensional fluorinated graphene: Synthesis, structures, properties and applications. Adv. Sci..

[54] Robinson J.T., Burgess J.S., Junkermeier C.E., Badescu S.C., Reinecke T.L., Perkins F.K., Zalalutdniov M.K., Baldwin J.W., Culbertson J.C., Sheehan P.E. (2010). Properties of fluorinated graphene films. Nano lett..

[55] Chantharasupawong P., Philip R., Narayanan N.T., Sudeep P.M., Mathkar A., Ajayan P.M., Thomas J. (2012). Optical power limiting in fluorinated graphene oxide: An insight into the nonlinear optical properties. J. Phys. Chem. C.

[56] Thiruppathi A.R., Sidhureddy B., Keeler W., Chen A. (2017). Facile one-pot synthesis of fluorinated graphene oxide for electrochemical sensing of heavy metal ions. Electrochem. Commun..

[57] Rui G., Zdeněk S., Filip Š., Zbyněk J., Martin P. (2015). Electrochemical fluorographene: Hybrid electrocatalysis of biomarkers, hydrogen evolution, and oxygen reduction. Chem. Eur. J..

[58] Damien D., Sudeep P.M., Narayanan T.N., Anantharaman M.R., Ajayan P.M., Shaijumon M.M. (2013). Fluorinated graphene-based electrodes for high performance primary lithium batteries. RSC Adv..

[59] Romero-Aburto R., Narayanan T.N., Nagaoka Y., Hasumura T., Mitcham T.M., Fukuda T., Cox P.J., Bouchard R.R., Maekawa T., Kumar D.S. (2013). Fluorinated graphene oxide; a new multimodal material for biological applications. Adv. Mater..

[60] Zhao F.-G., Zhao G., Liu X.-H., Ge C.-W., Wang J.-T., Li B.-L., Wang Q.-G., Li W.-S., Chen Q.-Y. (2014). Fluorinated graphene: Facile solution preparation and tailorable properties by fluorine-content tuning. J. Mater. Chem. A.

[61] Boopathi S., Narayanan T.N., Senthil Kumar S. (2014). Improved heterogeneous electron transfer kinetics of fluorinated graphene derivatives. Nanoscale.

[62] Jayaramulu K., Datta K.K.R., Rösler C., Petr M., Otyepka M., Zboril R., Fischer R.A. (2016). Biomimetic superhydrophobic/superoleophilic highly fluorinated graphene oxide and zif-8 composites for oil–water separation. Angew. Chem. Int. Ed..

[63] Mongay C., Cerda V. (1974). Britton–Robinson buffer of known ionic strength. Anal. Chim. Acta.

[64] Marcano D.C., Kosynkin D.V., Berlin J.M., Sinitskii A., Sun Z., Slesarev A., Alemany L.B., Lu W., Tour J.M. (2010). Improved synthesis of graphene oxide. ACS Nano.

[65] Iakovlev V.Y., Sklyueva Y.A., Fedorov F., Rupasov D., Kondrashov V., Grebenko A., Mikheev K., Gilmutdinov F., Anisimov A., Mikheev G. (2018). Improvement of optoelectronic properties of single-walled carbon nanotube films by laser treatment. Diam. Relat. Mater..

[66] Karikalan N., Karthik R., Chen S.-M., Velmurugan M., Karuppiah C. (2016). Electrochemical properties of the acetaminophen on the screen-printed carbon electrode towards the high performance practical sensor applications. J. Colloid Interface Sci..

[67] Liu Z., Xu J., Yue R., Yang T., Gao L. (2016). Facile one-pot synthesis of Au–PEDOT/rGO nanocomposite for highly sensitive detection of caffeic acid in red wine sample. Electrochim. Acta.

[68] Giacomelli C., Ckless K., Galato D., Miranda F.S., Spinelli A. (2002). Electrochemistry of caffeic acid aqueous solutions with pH 2.0 to 8.5. J. Brazil. Chem. Soc..

[69] Nematollahi D., Shayani-Jam H., Alimoradi M., Niroomand S. (2009). Electrochemical oxidation of acetaminophen in aqueous solutions: Kinetic evaluation of hydrolysis, hydroxylation and dimerization processes. Electrochim. Acta.

[70] Palanisamy S., Thangavelu K., Chen S.-M., Thirumalraj B., Liu X.-H. (2016). Preparation and characterization of gold nanoparticles decorated on graphene oxide@ polydopamine composite: Application for sensitive and low potential detection of catechol. Sens. Actuators B: Chem..

[71] Teymourian H., Salimi A., Khezrian S. (2013). Fe_3_O_4_ magnetic nanoparticles/reduced graphene oxide nanosheets as a novel electrochemical and bioeletrochemical sensing platform. Biosens. Bioelectron..

[72] Govindhan M., Amiri M., Chen A. (2015). Au nanoparticle/graphene nanocomposite as a platform for the sensitive detection of NADH in human urine. Biosens. Bioelectron..

[73] Bard A.J., Faulkner L.R., Leddy J., Zoski C.G. (2000). Electrochemical Methods: Fundamentals and Applications.

[74] Leite F.R.F., Santos W.d.J.R., Kubota L.T. (2014). Selective determination of caffeic acid in wines with electrochemical sensor based on molecularly imprinted siloxanes. Sens. Actuators B Chem..

[75] Diaconu M., Litescu S.C., Radu G.L. (2010). Laccase–MWCNT–chitosan biosensor—A new tool for total polyphenolic content evaluation from in vitro cultivated plants. Sens. Actuators B Chem..

[76] ElKaoutit M., Naranjo-Rodriguez I., Temsamani K.R., Hernández-Artiga M.P., Bellido-Milla D., Cisneros J.L.H.-H.d. (2008). A comparison of three amperometric phenoloxidase–Sonogel–Carbon based biosensors for determination of polyphenols in beers. Food Chem..

[77] Fernando d.S.L., Nelson R.S., Paulino O.H. (2008). Determination of caffeic acid in red wine by voltammetric method. Electroanalysis.

[78] Blasco A.J., González M.C., Escarpa A. (2004). Electrochemical approach for discriminating and measuring predominant flavonoids and phenolic acids using differential pulse voltammetry: Towards an electrochemical index of natural antioxidants. Anal. Chim. Acta.

[79] Del Alamo Sanza M., Domınguez I.N., Cárcel L.C., Gracia L.N. (2004). Analysis for low molecular weight phenolic compounds in a red wine aged in oak chips. Anal. Chim. Acta.

[80] Rodríguez-Delgado M.-Á., González-Hernández G., Conde-González J.-E.a., Pérez-Trujillo J.-P. (2002). Principal component analysis of the polyphenol content in young red wines. Food Chem..

