# Supplementary material for: Sensitive Electrochemical Detection of Caffeic Acid in Wine Based on Fluorine-Doped Graphene Oxide
Source: Sensors (Basel). 2019 Apr 3;19(7):1604. doi: 10.3390/s19071604 (PMC6480299; doi:10.3390/s19071604)
Supplement: Supplementary file 1 [file sensors-19-01604-s001.pdf]

Sensitive electrochemical detection of caffeic acid in wine based on fluorine-doped graphene oxide

Venkatesh S. Manikandan [1,2], Boopathi Sidhureddy [1], Antony Raj Thiruppathi [1] and Aicheng Chen [1,*]

[1] Electrochemical Technology Centre, Department of Chemistry, University of Guelph, 50 Stone Road E, Guelph, ON N1G 2W1, Canada; vmanikan@lakeheadu.ca (V.S.M.); bsidhure@uoguelph.ca (B.S.); athirupp@uoguelph.ca (A.R.T.)

[2] Department of Chemistry, Lakehead University, 955 Oliver Road, Thunder Bay, Ontario P7B 5E1, Canada

* Correspondence: aicheng@uoguelph.ca; Tel.: 1-519-8244120 Ext. 54764; Fax: 1-519-7661499

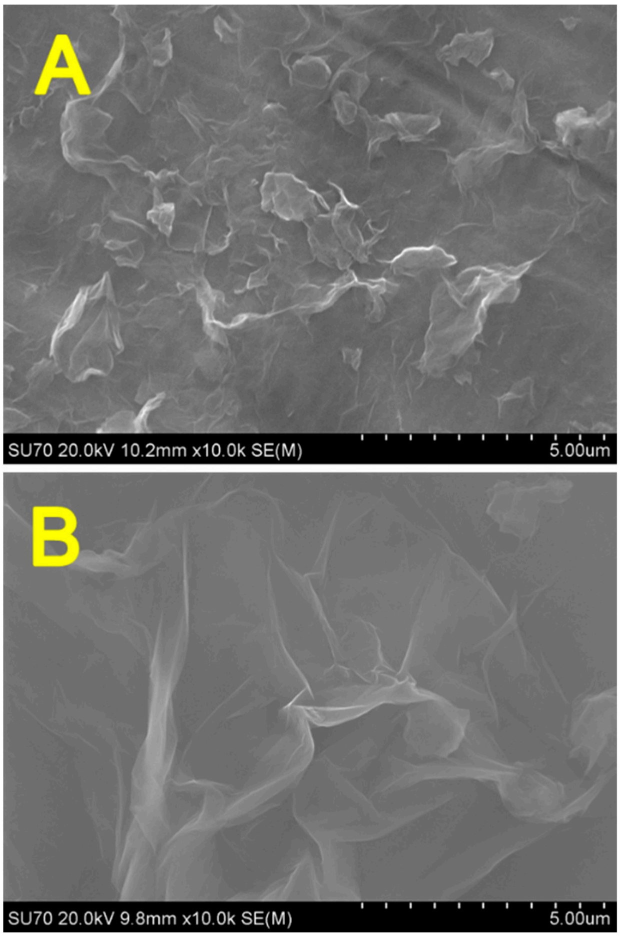

Figure S1. SEM images (low magnification) of the synthesized (A) graphene oxide (GO) and (B) fluorinated graphene oxide (F-GO)

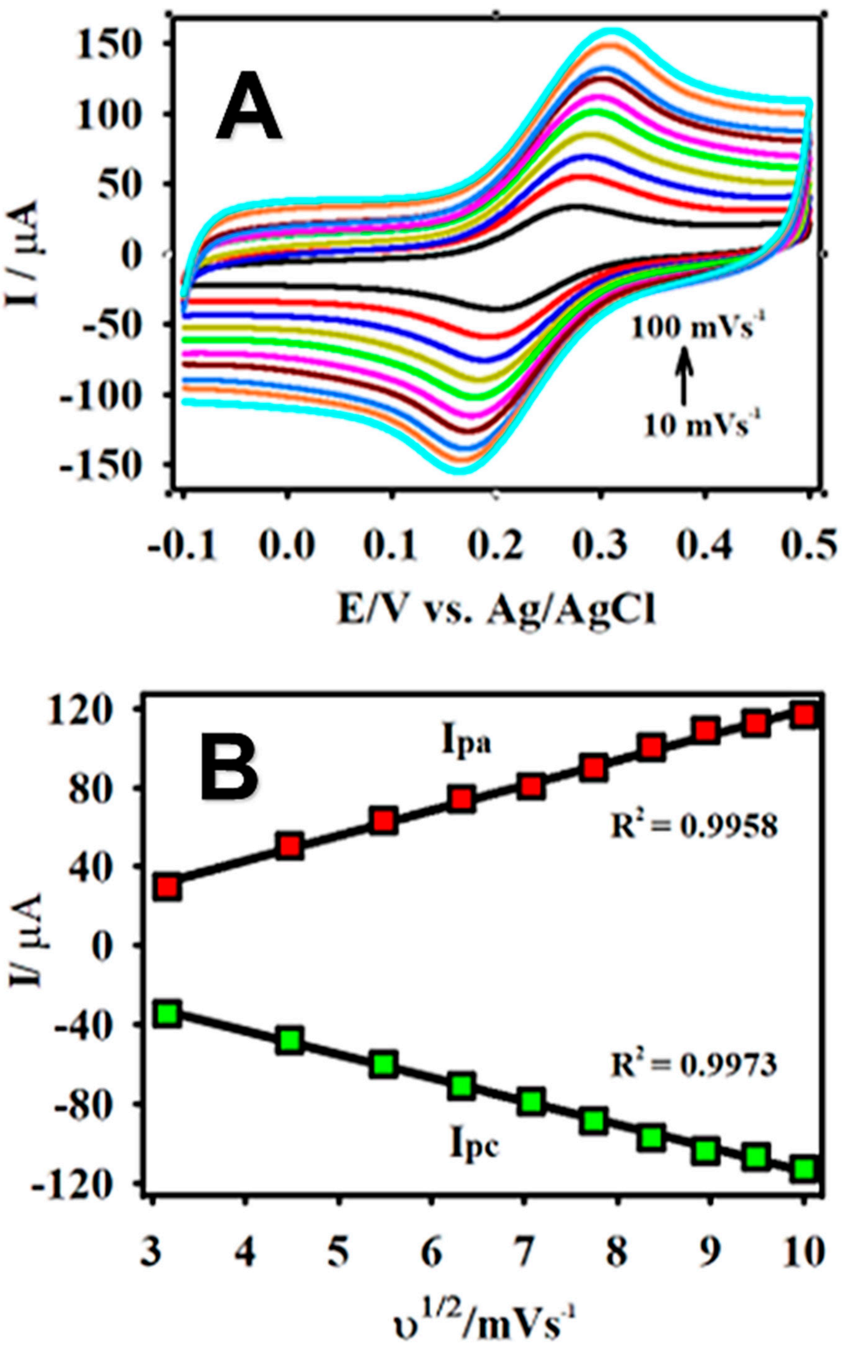

Figure S2. (A) CV curves of the F-GO/GCE recorded in the presence of 5 mM $[Fe(CN)_6]^{3-/4-}$ in 0.1 M KCl at various scan rates, ranging from 10 - 100 mV s^{-1}; (B) the corresponding plots of vs the anodic/cathodic peak current vs the square root of the scan rates.

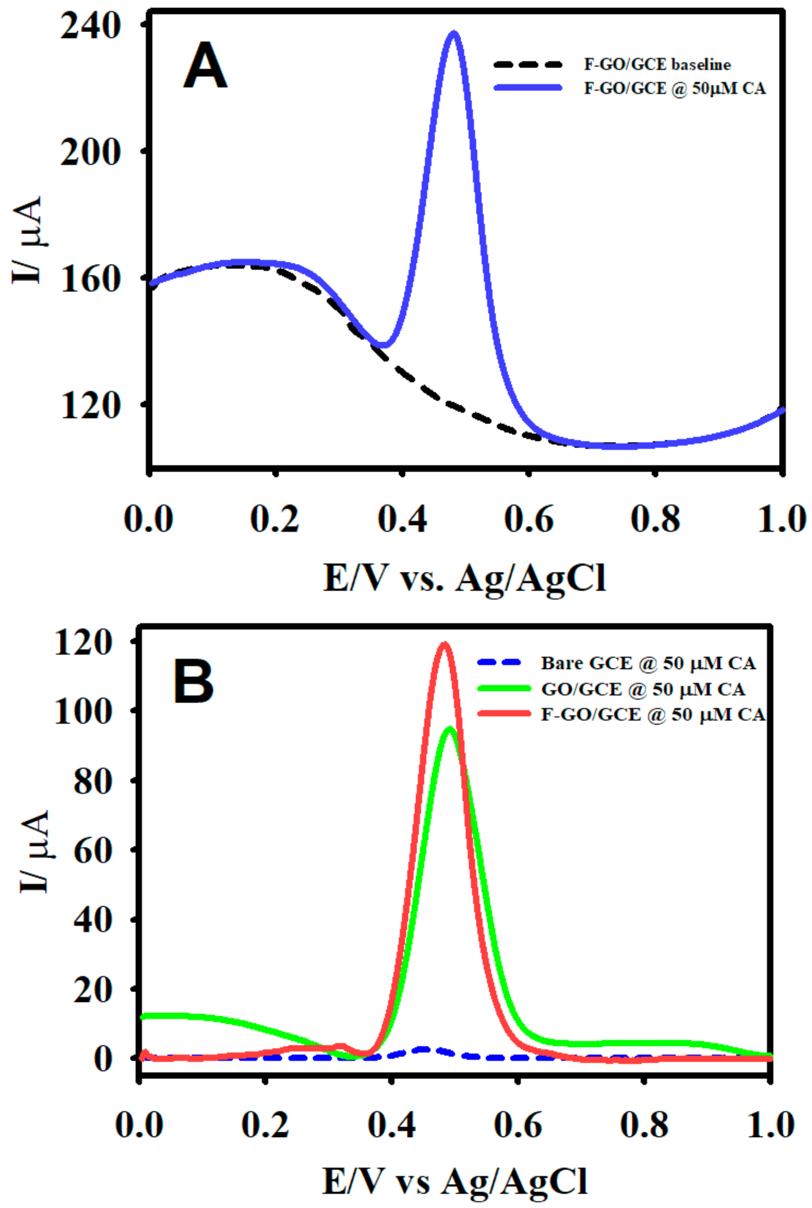

Figure S3. DPV response of F-GO/GCE recorded in the presence of 50.0 µM CA (blue line), in the absence of CA (black dashed line) (A); comparison of DPV response of bare GCE (blue dashed line), GO/GCE (green line) and F-GO/GCE (red line) recorded in the presence of 50.0 µM CA in 0.1 M B-R buffer solution (pH 2.65).

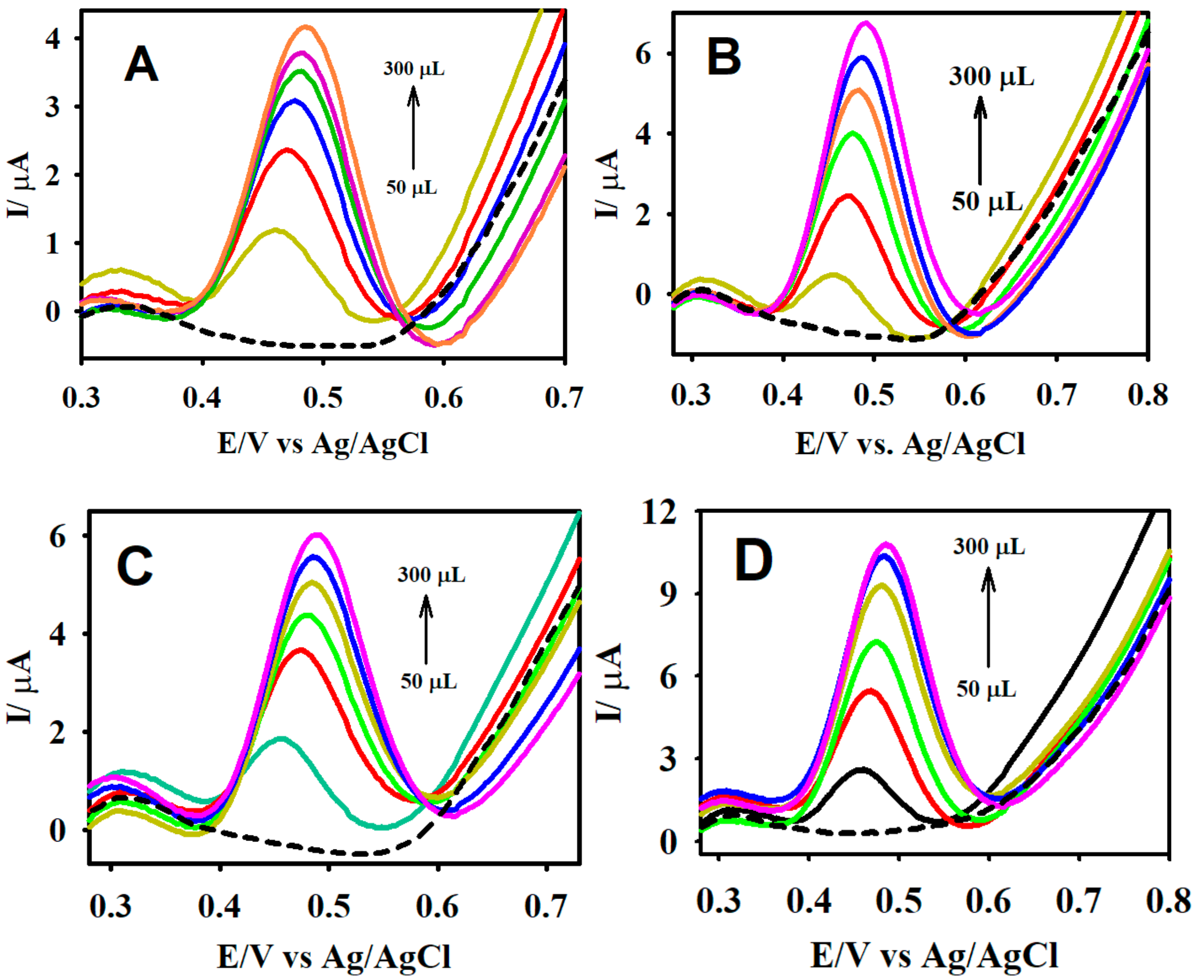

Figure S4. DPV response of F-GO/GCE for the detection of CA in different brands of red wine in 0.1 M B-R buffer solution (pH 2.65).

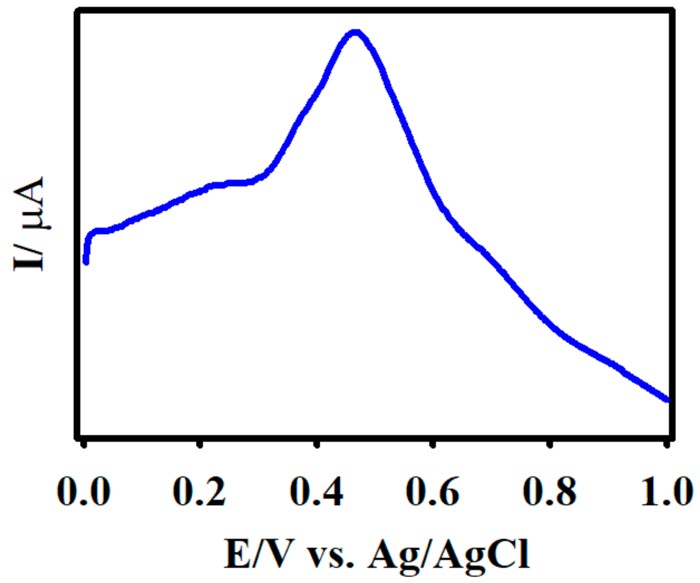

Figure S5. DPV response of the F-GO/GCE sensor toward the detection of CA directly in wine sample without an electrolyte medium
